# The Potential Mechanism of D-Amino Acids – Mitochondria Axis in the Progression of Diabetic Kidney Disease

**DOI:** 10.1016/j.ekir.2024.11.008

**Published:** 2024-11-14

**Authors:** Hoang Thuy Linh, Yusuke Nakade, Takashi Wada, Yasunori Iwata

**Affiliations:** 1 Department of Nephrology and Rheumatology, Kanazawa University, Japan; 2 Department of Clinical Laboratory, Kanazawa University, Japan

**Keywords:** D-amino acid, diabetic kidney disease, gut microbiota, mitochondria, oxidative stress, ROS production

## Abstract

Diabetic kidney disease (DKD) is a major complication of diabetes mellitus (DM) and stands out as the leading cause of end-stage renal disease worldwide. There is increasing evidence that mitochondrial dysfunction, including impaired mitochondrial biogenesis, dynamics, and oxidative stress, contributes to the development and progression of DKD. D-amino acids (D-AAs), which are enantiomers of L-AAs, have recently been detected in various living organisms and are acknowledged to play important roles in numerous physiological processes in the human body. Accumulating evidence demonstrates that D-AA levels in blood or urine could serve as useful biomarkers for reflecting renal function. The physiological roles of D-AAs are implicated in the regulation of cellular proliferation, oxidative stress, generation of reactive oxygen species (ROS), and innate immunity. This article reviews current evidence relating to D-AAs and mitochondrial dysfunction and proposes a potential interaction and contribution of the D-AAs–mitochondria axis in DKD pathophysiology and progression. This insight could provide novel therapeutic approaches for preventing or ameliorating DKD based on this biological axis.

### DKD and Mitochondria: Generalities

According to the International Diabetes Federation, approximately 537 million people aged between 20 and 79 years (10.5% of all adults in this age group) are living with DM. And, yet, it continues predicting that the total number of people with diabetes will increase to over 643 million by 2030 and 783 million adults aged 20 to 79 years by 2045.^1^ DKD is a major complication of DM, as well as the leading cause of end-stage renal disease worldwide.^2^^,^^3^ About 30% of individuals diagnosed with type 1 diabetes and 40% with type 2 diabetes advance to DKD.^4^ The overall mortality rate associated with DKD is approximately 30-fold greater than that observed in diabetic individuals without nephropathy, consequently imposing a substantial economic burden on both patients and the country.^5^

The pathogenic mechanisms of DKD are complex, multifactorial, and poorly understood. Hyperglycemia is the initiating factor that triggers structural and functional impairments to multiple organs, including the kidney. Conventional pathogenesis involves alteration in renal hemodynamics, ischemia, and glucose metabolism abnormalities–associated oxidative stress increases, inflammatory processes, and overactive renin-angiotensin-aldosterone system.^6^ Recent molecular and cellular biology studies explore novel fields of the pathophysiology of diabetes and DKD, including genetic and epigenetic regulation, mitochondria dysfunction, and podocyte autophagy.^7^ These mechanisms allow for more precise targeting in the treatment of DKD.

Mitochondria are specialized organelles with 2 distinct membranes and their own genome and bacterial-type ribosomes.^8^ They are present and play an essential role in the survival of all eukaryotic cells except mature erythrocytes. Known as the cell powerhouse, the mitochondria's most prominent function is generating cellular energy in the form of adenosine triphosphate (ATP) using a series of redox reactions via the electron transport chain system (oxidative phosphorylation), accounting for over 90% of the energy production in the human body. Moreover, mitochondria are involved in diverse biological processes, such as the generation of ROS, calcium signaling, metabolic networks, proliferation, cancer, and apoptosis.^9^ As one of the most metabolically active organs with high energy requirements, kidneys contain the second highest density of mitochondria in the body.^10^ Importantly, the mitochondria found in different cell types within the kidney exhibit heterogeneity in quantity, form, and physiological role. The proximal and distal tubules in the cortex are abundant in mitochondria, whereas the tubular cells of the collecting duct and loop of Henle in the medulla contain fewer mitochondria.^11^^,^^12^ The cortex is the main location of fatty acid oxidation and renal gluconeogenesis, whereas the medulla has higher rates of glycolysis.^13^ Podocytes have a moderate density of mitochondria and display a flexible phenotype utilizing both oxidative phosphorylation and glycolysis to balance their energy requirement.^14^, ^15^, ^16^

### Mitochondrial Impairment in DKD

Experimental models of DKD pointed out that mitochondrial dysfunction, including mitochondrial biogenesis, dynamics, and oxidative stress, precedes proteinuria and renal histological changes. For example, in the early stage (4 weeks after streptozotocin injection), mitochondrial fragmentation and decreased ATP content occurred in proximal tubular cells without albuminuria and specific glomerular pathology. After 8 weeks of diabetes, they progressed toward mitochondrial permeability transition pore opening, mitochondrial-generated oxidative stress, and glomerular alterations. Evidence of tubular injury associated with increased complex I-dependent oxidative phosphorylation and decreased ATP content was observed after 16 weeks.^17^ Patients with DKD have disrupted mitochondrial dynamics, uncoupling, oxidative damage, decreased mitochondrial respiratory capacity, decreased mitochondrial DNA content, and decreased antioxidant capacity.^18^ Although the link between mitochondrial dysfunction and DM has been observed for a long time, understanding the exact mechanisms by which mitochondrial dysfunction contributes to DKD development remains ambiguous. Two primary theories explain the pathological role of mitochondrial dysfunction in the kidney: one suggests increased mitochondrial oxygen consumption and superoxide production leading to oxidative damage; and the other proposes a general impairment of mitochondrial function and biogenesis, ultimately reducing ATP production.^19^^,^^20^

Oxidative stress (or oxidant-derived tissue injury) occurs when production of oxidants or ROS exceeds local antioxidant capacity. The ROS family includes highly reactive free oxygen radicals (such as superoxide anion, O2^•–^; and hydroxyl radical, ^•^OH) and the stable “diffusible” nonradical oxidants (hydrogen peroxide [H_2_O_2_]). Mitochondria are the primary source of ROS in cells, via the respiratory chain.^21^ The “unifying hypothesis” suggests that chronically driven glucose overproduction of mitochondrial ROS leads to cellular and eventual kidney failure. Increased mitochondrial ROS production has been demonstrated by the bulk of evidence in multiple mouse models of DKD, triggering inflammation, interstitial fibrosis, and apoptosis in DKD.^20^, ^21^, ^22^, ^23^, ^24^, ^25^, ^26^ However, recent *in vivo* and *ex vivo* studies have provided evidence of an alternative mechanism of mitochondrial involvement in DKD, associated with reduced mitochondrial superoxide.^27^ Based on these findings, the new theory of “mitochondrial hormesis” is proposed, where either elevated or reduced mitochondrial ROS levels, through a biphasic response, increase the risk of tissue damage.^28^ Although the superoxide anion radical is an important, primary form of ROS, it is short-lived in most cases, rapidly converted to H_2_O_2_, and cannot cross membranes because of its negative charge. H_2_O_2_, which has a long half-life and can move through the cell membrane, acts as a secondary cell signaling messenger to reach far effects.^29^ Perhaps, mitochondrial superoxide plays an indicator of altered mitochondrial function and a “starter” to initiate a cascade, including lipid peroxidation processes, damaging both membrane structure and function. It may also be responsible for the oxidation of key proteins involved in cellular metabolism and function, ultimately leading to the oxidation of nucleic acids.^27^^,^^28^ Mitochondrial oxidative damage-promoted ectopic lipid deposition, lipid peroxidation, impaired fatty acid oxidation, and sphingolipid homeostasis contributed to diabetic renal tubular injury. SS31, a mitochondria-targeted antioxidant, restored reprogramed lipid metabolism and protected tubular epithelial cell damage.^30^

Dynamic remodeling of mitochondrial networks by fission, fusion, and mitophagy promotes the maintenance of cellular function and survival under different physiological conditions. Excessive mitochondrial fission and elevated fragmented mitochondria are early features of kidney injury before the obvious clinical manifestations of DKD.^17^ If mitochondria are irreparably damaged, they are removed through mitophagy to prevent the production of ROS, supply raw materials for metabolic processes, and support mitochondrial biogenesis. Lower NDUFS4 expression, an accessory subunit of mitochondrial complex I, results in impaired complex I and respiratory supercomplex formation, significantly impacting podocyte bioenergetic capacity, cristae integrity, and mitochondrial morphology, thus promoting DKD progression.^31^ Enhancing the level of intracellular mitophagy has a considerable renoprotective impact on DKD. For example, treatment with a mitochondrial antioxidant, MitoQ protected against DKD by upregulating the levels of mitophagy via Nrf2/PTEN-induced putative kinase protein 1.^32^, ^33^, ^34^

Mitochondrial biogenesis facilitates the close collaboration between the nuclear and mitochondrial genomes to replicate mitochondrial DNA, produce new and functional mitochondria, ensure mitochondrial stability, and regulate normal metabolic processes within cells.^35^ Urine metabolomics indicated reduced metabolites linked to mitochondrial metabolism, implying inhibited mitochondrial function in patients with DKD. Kidney biopsies also revealed decreased mitochondrial DNA; protein expression; and peroxisome proliferator-activated receptor γ coactivator-1α gene expression, a key regulator of mitochondrial biogenesis.^36^ Loss of renal peroxisome proliferator-activated receptor γ coactivator-1α is an important step in the pathogenesis of DKD, and therapies that enhance the activity of peroxisome proliferator-activated receptor γ coactivator-1α are associated with renoprotection and mitochondrial biogenesis.^37^, ^38^, ^39^, ^40^

Mitochondria are also key organelles of calcium signaling. Ca^2+^ is an important intracellular second messenger, controlling normal oxidative respiration and ATP production by regulating the activity of TCA cycle rate-limiting enzymes^41^ and ATP synthase.^42^ Mitochondria are spatially and functionally connected with the endoplasmic reticulum through “mitochondria-associated membranes” to maintain mitochondrial Ca^2+^ homeostasis. Activation of the TRPV1 channel by capsaicin reduced mitochondria-associated membranes formation and Ca^2+^ transport from the endoplasmic reticulum to mitochondria, thereby decreasing high glucose–induced mitochondrial damage in podocytes.^43^

### D-AAs in Kidney Disorders

D-AAs have traditionally been regarded as unnatural AAs. However, owing to the technological advances and improvements in detection methods for chiral AAs, research in the mid–20th century revealed the presence of D-AAs in mammals.^44^ Its main origins are food intake, intestinal microbiota, and intrinsic biosynthesis. The initial findings and most rapid progress in this area originate from research in the field of neuroscience. It is related to N-methyl-d-aspartate (NMDA) receptor, an important ion channel in neurological tissues. D-serine, D-alanine, D-aspartate, and D-glutamic acid are important agonists of NMDA receptors, so researching these AAs is crucial for comprehending the development of diseases directly involving the NMDA receptor such as Alzheimer's Disease and schizophrenia.^45^^,^^46^

The investigation of D-AAs in the kidneys (summary in Table 1)^47^, ^48^, ^49^, ^50^, ^51^, ^52^, ^53^, ^54^, ^55^, ^56^, ^57^, ^58^, ^59^, ^60^, ^61^, ^62^ can be traced back to 1987. Nagata *et al.*^47^ showed an increase in the total amount of plasma D-AAs in older individuals or those with renal diseases. Moreover, it correlated positively with serum creatinine, *β*_2_-microglobulin levels, and glomerular filtration rate.^47^ Subsequent studies reached similar conclusions^48^^,^^49^; however, at that time, no precise methods were available to detect trace amounts of D-AAs. With the advanced ability to accurately measure D-AAs using 2-dimensional high-performance liquid chromatography, research on the role of D-AAs in the kidneys has resumed and progressed rapidly.

**Table 1:** D-AAs and kidney function

	Findings	Reference
Clinical studies		
Various renal diseases	Higher total plasma D-AA levels in an elderly and renal disease groups and correlated to the serum creatinine, *β*2-microglobulin, and glomerular filtration rate	^47^
	Serum D-Ser of all patients with renal disorders and correlated to creatinine levels	^48^
	Serum D-Pro of all patients undergoing hemodialysis and with nephrotic syndrome	
	Urinary DAO index correlated with changes in urinary microalbumin and N-acetyl-beta-glucosaminidase.	^49^
CKD	Association of plasma D-Ser, D-Pro, and D-Asn with eGFR; D-Ala and D-Pro with age; D-Asp and D-Pro with DM; D-Ser and D-Asn with the progression of CKD	^50^
	Blood D-Ser correlated with the actual glomerular filtration ratio	^51^
	Urinary dynamics of D-Ser reflected the presence of CKD	
	% values of D-Aas, especially D-Asn and D-Ser correlated with kidney function	^52^
	Increased plasma levels of D-Asn, D-Ser, D-Ala, D-Pro	^53^
	Decreased urine levels of D-Asn and D-Ser	
	Increase intrabrain D-Asn levels	
	Decreased plasma L-Ser levels with declined cognitive function	
	Increased plasma D-Ala and D-Ser in CKD	^54^
	Higher plasma D-Ala in CKD with DM	
	Increased salivary Streptococcus in CKD with DM; positively correlated with plasma D-Ala levels	
	Higher plasma D-Asn and the D/L-Ser associated with serum creatinine and cystatin C in pediatric patients	^55^
IgA nephropathy	Higher urine levels of D-Ser and lower levels of other D-AAs. Urine D-Asp and D-Cys levels and blood albumin levels, urine D-His and D-Asp levels and fasting hyperglycemia, urine D-Thr levels and neutrophils and white blood cell counts, and urine D-ser levels and serum creatinine levels.	^56^
Peoples with therosclerotic risk factors	Urinary D-Ser predicted deteriorating renal function	^51^
HIV	%D-AAs were correlated with kidney function in both with or without HIV infection	^57^
Schizophrenia	Reduced kidney function decline with risperidone treatment (inhibiting DAO activity)	^58^
Murine models and clinical data		
AKI	D-Ser suppressed hypoxia-induced tubular damage and promoted posthypoxic tubular cell proliferation.	^59^
	The oral administration of D-Ser improved renal I/R-induced injuries directly or through modulating gut microbiota.	
	Increased D-Ser/L-Ser ratio in patients with AKI; inversely correlated with renal function	
	D-Ala/NMDAR inhibit ROS production and improve mitochondrial membrane potential, resulting in reduced TEC necrosis by hypoxic stimulation	^60^
	Oral administration of D-Ala ameliorated kidney injury after I/R induction	
	Increased plasma D-Ala levels and reflects renal function in patients with AKI.	
Cisplatin-induced AKI	Cisplatin-induced damage of proximal tubules accompanies Asc-1 induction in tubules and collecting ducts and leads to serum D-Ser accumulation with a positive correlation to serum creatinine	^61^
Kidney remodeling	Increased plasma D-Ser in human living kidney donors after nephrectomy	^62^
	Treatment of D-Ser at a low dose promotes the enlargement of remnant kidney in mouse model. D-Ser activates the cell cycle for tissue remodeling through an mTOR-related pathway.	

AKI, acute kidney injury; CKD, chronic kidney disease; D-AA, D-amino acid; D/L-Ser, D/L-serine; D-Ala, D-alanine; D-Pro, D-proline; D-Asn, D-asparagine; D-Thr, D-threonine; DAO, D-amino acid oxidase; DKD, diabetic kidney disease; DM, diabetes mellitus; eGFR, estimated glomerular filtration rate; NMDAR, N-methyl-d-aspartate receptor; TEC, tubular epithelial cell.

Initially, a series of studies discovered the clinical significance of D-AAs in chronic kidney diseases (CKDs). Notably, D-serine could serve as a powerful biomarker for both early diagnosis and prognosis of CKD.^50^, ^51^, ^52^^,^^63^ D-serine, D-proline, and D-asparagine levels showed a strong correlation with kidney function (estimated glomerular filtration rate).^50^^,^^52^ In addition, D-alanine and D-proline levels were linked to age, and D-aspartate and D-proline levels were associated with DM.^50^ Morishita *et al.*^55^ found that D-asparagine and the D-to-L-serine ratio remained unchanged regardless of body size in children and are unaffected by glucocorticoid treatment in mice. Therefore, plasma D-asparagine and the D-to-L-serine ratio may serve as reliable endogenous biomarkers for evaluating renal function or detecting CKD during growth or steroid therapy.^55^ In patients with CKD, Iwata *et al.*^53^ observed increased levels of plasma D-serine and D-asparagine, and intra-brain D-asparagine. However, reduced levels of brain and plasma L-serine rather than D-AAs, are associated with impaired cognitive function in patients with kidney dysfunction.^53^ In a larger CKD population, plasma D-alanine and D-serine levels were increased. Specifically, D-alanine levels were higher in patients with both CKD and DM than in those without DM.^54^ Altered D-AA levels were also observed in acute kidney injury (AKI),^59^, ^60^, ^61^^,^^64^ IgA nephropathy,^56^ and HIV infection,^57^ confirming that D-AAs could serve as a powerful biomarker to reflex renal function.

A major concern in using D-AAs as biomarkers is their lack of specificity, because changes in D-AA levels are not always exclusive to specific disease conditions. For example, increased blood D-serine levels have been observed in patients with AKI,^59^ CKD,^53^ and various neurological disorders.^46^ Similarly, decreased urinary D-asparagine levels are seen not only in CKD^53^ but also in the presence of glioblastoma.^65^ Although most studies have found a link between D-AA levels and renal function, the evidence is insufficient to conclusively suggest that these levels are specific to any particular disease. Several questions need to be addressed to determine whether these changes are disease-specific or nonspecific. First, the gut microbiota, a primary source of D-AAs, function as a vital bridge to other organs. Emerging evidence has linked gut dysbiosis to various diseases, which are directly related to altered D-AA levels in the body.^66^ Second, there is a lack of knowledge about D-AA transportation, metabolism, and function. The mechanisms involved in D-AA transport have not yet been fully understood. Although D-AA-metabolizing enzymes, such as D-AA oxidase (DAO), serine racemase, and D-aspartate oxidase (DDO) were identified earlier, understanding their distribution and activity in specific diseases remains limited and is still being explored. The presence, functions, and roles of D-AAs in mammalian systems have only been discovered in the last 2 decades and are still actively under investigation. A comprehensive understanding of the origin, metabolism, and functions of D-AAs is urgently needed to determine their specificity and sensitivity as biomarkers.

Furthermore, D-AAs are bioactive substances, and the physiologic functions of several D-AAs have been identified to date. Specifically, D-serine modulates neural signaling in the cerebral cortex and is involved in memory and learning processes.^45^^,^^46^ D-aspartate, notably abundant in the central nervous, neuroendocrine, and endocrine systems, plays a physiological role in regulating hormone secretion and steroidogenesis.^67^^,^^68^ Our research group indicated that D-serine protects against AKI either directly or by modifying the gut microbiota.^64^ Moreover, Hesaka *et al.*^62^ found the relationship between D-serine and the cellular proliferation of proximal tubules via the mTOR pathway in kidney remodeling. D-alanine demonstrates a protective effect against AKI by inhibiting the production of ROS and enhancing matrix metalloproteinases, leading to decreased necrosis of tubular epithelial cells under hypoxic conditions.^60^

### D-AAs, Mitochondria, and Potential Application in DKD

To date, only a limited number of studies have examined the effects of D-AAs on mitochondrial metabolism and function, as well as specific roles of D-AAs in DKD. However, as discussed earlier, hyperglycemia modulates mitochondrial impairment through phosphorylation and oxidation of mitochondrial proteins, production of ROS, and decreased antioxidant capacity. Chronic hyperglycemia initiates cell damage; however, as the disease progresses, a complex interplay of factors perpetuates and exacerbates ROS production and oxidative stress. Disturbed metabolism, altered hemodynamics, hypoxia, inflammation, the formation of advanced glycation end products (AGEs), gut microbiome, or diet contribute to this process. These factors create a vicious cycle that sustains oxidative stress, leading to progressive tissue damage in diabetic complications, including DKD.^69^^,^^70^ The impact of D-AAs on these processes represents a promising target and application for DKD.

DAO and DDO are stereospecific degrative enzymes that metabolize D-AAs. DAO acts on nonpolar D-AAs such as D-alanine and D-serine and hydrophobic, bulky D-AAs such as D-tyrosine, D-tryptophan, D-phenylalanine; whereas DDO metabolizes acidic D-AAs, particularly D-aspartate, D-glutamic acid, and NMDA. Recently, novel mitochondrial proteins such as 9030617O03Rik and pLG72 have been reported to be associated with D-glutamic acid and D-serine levels, but detailed metabolic and functional pathways still need to be clarified.^71^^,^^72^ Both DDO and DAO catalyze the oxidative deamination of D-AAs, spontaneously producing H_2_O_2_.^73^ High levels of DAO expression and enzyme activity are found in the mammalian kidney, liver, brain, and to a lesser extent in leukocytes, small intestine, epididymis, preputial and adrenal glands, although the expression pattern varies among species.^74^ As mentioned above, H₂O₂, a relatively stable and long-lived ROS molecule, has both positive and negative effects depending on its level and the cell type under investigation.^75^ H_2_O_2_ causes oxidative stress and eventual cell death at high levels.^76^ D-AAs could exhibit physiological impacts either directly or by modulating the activity of DAO, DDO, or NMDA receptors.

An *in vitro* study in rat liver mitochondria reported that D-serine, D-alanine, D-methionine, D-aspartate, D-tyrosine, and D-arginine inhibit mitochondrial function by increasing free radical production. However, in the presence of oxidative stress, D-AA addition resulted in a protective effect on state 3 respiration.^77^ Yap *et al.*^78^ demonstrated that D-serine and D-alanine induced inflammation via distinct mechanisms in human liver cancer cells (HepG2). D-serine may cause apoptosis through matrix metalloproteinase collapse and elevated levels of TNF-α and activated NF-κB. In contrast, D-alanine increased NF-κB activation and production of TNF-α and IL-8, but did not induce significant apoptosis.^78^ DAO overexpression has been reported to promote DNA damage–induced senescence through ROS production; however, whether DAO activity itself or specific D-AAs modulate DAO during senescence is still unclear.^79^ The administration of an adequate concentration of D-serine reduced kidney injury, and a higher concentration of D-serine, such as 80 mM, augmented kidney damage in I/R-induced AKI mice.^59^ Another study discovered the protective effect of D-alanine/NMDA receptor signaling in mitochondria. In human kidney (HK-2) cells, treatment with 1-100 μM D-alanine reduced mitochondrial fragmentation, production of ROS, and mitochondrial membrane depolarization under hypoxic conditions.^60^ The beneficial effects of D-Alanine were inhibited by Atpenin A5, a mitochondrial complex II inhibitor, and MK-801, an NMDA receptor inhibitor. Moreover, an 80 mmol dose of D-alanine decreased the number of fragmented and swollen mitochondria, offering renoprotection in mice with I/R-induced AKI.^60^ Clearly, the dose and specific condition impact the efficacy of D-AAs. D-alanine was reported to induce gluconeogenesis in the kidney through the circadian transcriptional network.^80^

D-arginine protects cells against radical-induced myocardial injury by exerting a superoxide anion (O2-) scavenging activity^81^ and an antiapoptotic action,^82^ and displays central stimulant properties.^83^ Excessive L-arginine concentrations have been associated with increased ROS leading to neurotoxicity and senescence in the central nervous system. D-arginine exhibited its neuroprotective effects by neutralizing the excessive L-arginine concentration in the intercellular space.^84^ D-arginine reduces the hypertensive response in rats dosed with angiotensin II and improves acetylcholine-induced aortic relaxation *ex vivo,* without significantly affecting the antioxidant status of the plasma.^85^ This implies that the observed antihypertensive action is based on other unknown mechanisms. Methylglyoxal, a reactive dicarbonyl molecule, generated as a byproduct in the metabolic processes of glucose, fatty acids, and AAs to different degrees, is a major precursor for the formation of AGEs.^86^ The detrimental effects of the formation of AGEs are strongly implicated in the pathogenesis of conditions such as vascular complications of diabetes, DKD, and aging.^87^ Excessive AGEs and dicarbonyl compounds also exhibit deleterious impacts on mitochondria function.^88^^,^^89^ D-arginine and L-arginine attenuate methylglyoxal- and high glucose–induced endothelial dysfunction, oxidative stress, and AGE formation. Of interest is that D-arginine becomes a more favorable option for pharmacokinetic, pharmacodynamic properties, and therapeutic potential, thereby specifically inhibiting the harmful repercussions of methylglyoxal on various functional and biochemical processes.^90^ Only D-arginine was transported across the mitochondrial membrane in response to an electric potential,^91^ supposing its involvement in the metabolic and functional activities of mitochondria. Deeper mechanisms, including specific pathways, molecules, or interacting targets of D-arginine should be urgently investigated to provide a promising therapeutic strategy for mitochondrial disorders and DKD.

D-cysteine protects neurons from oxidative stress or renal ischemia-reperfusion injury by converting to hydrogen sulfide (H_2_S). Once absorbed from the gastrointestinal tract and into the bloodstream, D-cysteine can be rapidly metabolized to H_2_S through 3-mercaptopyruvate sulfurtransferase and DAO.^92^ H_2_S acts as a signaling molecule and cytoprotectant, reducing inflammation, preserving mitochondrial function, and antiapoptosis in neurons, heart, and kidney injuries.^93^, ^94^, ^95^ Another study by Xiang *et al.*^96^ indicated the protective effect of D-cysteine in CKD. D-cysteine can enhance cell viability and mitochondrial function in hypoxia-induced injury HK-2 cells. H_2_S generated from D-cysteine prevented oxidative stress and inhibited inflammation, thus slowing down the development of renal fibrosis in adenine-induced CKD mice.^96^ Both studies demonstrated that D-cysteine is more effective than L-cysteine, providing greater protection against kidney injury.^92^^,^^96^ D-/L-cysteine supplementation protected hypertension and kidney damage in spontaneously hypertensive rats treated with high-salt through the H_2_S-generating 3-mercaptopyruvate sulfurtransferase-DAO pathway. They not only reduced oxidative stress but also regulated the renin-angiotensin system by increasing renal AT2R protein levels and decreasing renal renin mRNA levels.^97^

### Microbiota, a Source of D-AAs: the Key Player in the Pathogenesis of DKD

Gut dysbiosis, bacterial diversity variations, microbial composition changes, and a “leaky gut” in patients with DKD have been reported.^98^, ^99^, ^100^ An imbalanced microbiome and injured intestinal barrier can lead to the leakage of bacteria and/or bacterial products from the lumen, known as bacterial translocation. This causes a chronic inflammation that gradually intensifies kidney damage, creating a vicious cycle in which dysbiosis and renal dysfunction progressively worsen.^101^

As the main source of D-AAs, bacteria produce a largely distinct group of D-AAs that are incorporated into the cell wall and also released as free D-AA in the gut lumen.^102^^,^^103^ The renoprotective effect of gut microbiota-derived D-serine was reported in AKI.^59^ D-alanine, D-serine, D-cysteine, and D-methionine may contribute to regulating the gut microbiota composition, as well as exhibiting antioxidant and antiinflammatory activities in experimental models of intestinal damage.^104^, ^105^, ^106^, ^107^, ^108^, ^109^ Intestinal macrophages recognize D-AAs to enhance survival and differentiate B cells against pathogens.^110^^,^^111^ D-tryptophan supplementation reestablished a healthy microbial community by increasing intestinal bacterial diversity and reduced Th2 cell differentiation, significantly preventing the full development of allergic airway disease.^112^ H₂O₂ could act as a barrier between the gut lining and bacteria, preventing microbes from coming into proximity to the intestinal surface.^113^ Sasabe *et al.*^114^ suggested that intestinal epithelial cells release DAO into the lumen to oxidize mucosal D-AAs, subsequently generating H₂O₂ to protect the proximal intestinal mucosa by killing entero-pathogens. Systemic H_2_O_2_-generated neutrophil DAO has been reported to assist in the defense against pathogens.^110^^,^^115^^,^^116^ Targeting D-AA supplementation or modulating DAO activity may help maintain a healthy gut microbiome, protect against bacterial translocation, and support immune homeostasis. This approach could be a promising therapeutic strategy to delay the pathogenesis of DKD.

Host mitochondria can import certain bacterial proteins because of the similarity in targeting sequences between bacterial and mitochondrial proteins.^117^ Mitochondrial dysfunction and mitochondrial DNA impact gut microbiome communities.^118^, ^119^, ^120^, ^121^ In contrast, the microbiota can directly regulate mitochondrial biological processes via key transcription factors, coactivators, and enzymes.^117^^,^^122^ While the direct effects of D-AAs on mitochondrial function are still being investigated, there is a suggestion of an association between D-AAs, DAO activity, H_2_O_2_ levels, oxidative stress, and inflammation because of the antioxidant and antiinflammatory effects of D-AAs. More importantly, D-AAs may indirectly impact mitochondria by modulating gut microbiota composition and the intestinal immune environment. This interaction is bidirectional: mitochondrial homeostasis can in turn, regulate gut microbiota, which subsequently influences D-AA levels.

### Therapeutic Strategies and Future Perspectives

To date, numerous areas remain unidentified in the pathological mechanisms of D-AAs, mitochondrial dysfunctions, and even DKD. Building upon the above summary, we propose that an association between D-AAs and mitochondria could be involved in the pathophysiology and progression of DKD (Figure 1). D-AAs and their associated metabolic enzymes contribute to the production of ROS, especially long-lasting H_2_O_2_ (D-alanine, D-serine),^78^ lipid peroxidase (D-methionine, D-cysteine),^107^^,^^109^ AGEs (D-arginine),^90^ cytoprotective H_2_S (D-cysteine),^107^ and other unknown mechanisms. These processes continue to play a role in modulating inflammation, apoptosis, cell proliferation, cellular senescence, fibrosis, and further. Some novel mitochondrial proteins such as 9030617O03Rik and pLG72, are correlated with D-AA levels, but the underlying mechanisms remain unclear and need to be investigated further.^71^^,^^72^ Moreover, the correlation between D-AAs and the gut microbiota axis holds considerable significance. D-AAs are produced by the gut microbiota, and along with DAO activity, they participate in a regulatory feedback mechanism that influences gut microbiota diversity and contributes to maintaining immune homeostasis in the intestinal environment (such as D-serine,^59^^,^^108^ D-alanine,^104^^,^^106^ D-tryptophan,^112^ D-methionine,^109^ and D-cysteine^107^). D-AAs and mitochondria may interact with each other directly or indirectly by modulating the gut microbiome community. This complex relationship between D-AAs, mitochondrial function, and the gut microbiota underscores the interconnectedness of microbial metabolites, cellular health, and immune regulation in the gut.

**Figure 1:**
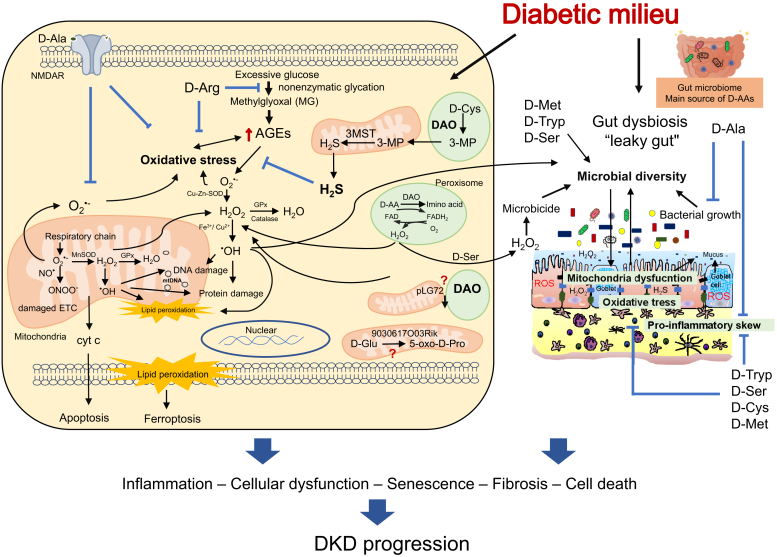
A potential biological interaction of D-AAs and mitochondria in DKD pathogenesis. AGEs, advanced glycation end products; ETC, electron transport chain; D-AA, D-amino acid; D-Ala, D-alanine; D-Arg, D-arginine; D-Cys, D-cysteine; D-Met, D-methionine; D-Tryp, D-tryptophan; D-Ser, D-serine; DAO, D-amino acid oxidase; DKD, diabetic kidney disease; NMDAR, N-methyl-d-aspartate receptors; ROS, reactive oxygen species; SOD, superoxide dismutase; 3MP, 3-mercaptopyruvate; 3MST, 3-mercaptopyruvate sulfurtransferase.

With significant advances in understanding the signaling roles of D-AAs in various neuropathologies, the use of D-AAs in brain disorders has widened, gaining attention in both academic research and pharmaceutical development worldwide.^123^

In the field of kidney research, present studies have demonstrated the role of D-AAs as a biomarker to reflex renal function. Of course, further clinical investigations across diverse disease conditions are necessary to validate their specificity in diagnostic and prognostic roles. Delving into the mechanisms of the D-AAs - mitochondrial axis should be intensified to uncover efficacy of therapeutic approaches to delay DKD progression. The treatment of DKD has evolved significantly beyond simply controlling blood glucose; current approaches now employ a multifaceted strategy targeting various pathophysiological mechanisms. It is crucial to remember that the advantages or drawbacks of D-AAs depend on dosage, route of administration, intervention duration, organ type, and specific disease conditions. Strategies using probiotic bacteria or fermented food (containing beneficial D-AAs) should be considered.

Therapeutic aspects have been progressing, concentrating on the effects of D-AA intake on AA metabolism and kidney function in humans. Short-term D-alanine administration was well-tolerated in healthy adults, maintaining estimated glomerular filtration rate levels without serious adverse events and having no impact on other kidney function markers. The demonstrated potential efficacy and safety profile of D-alanine will be valuable in future clinical trials involving patients with CKD.^124^ D-AAs could be supplemented through diet. Sake lees, which contain dietary fiber, bacteria, and D-AAs (D-alanine and D-serine), are added to the standard CKD dietary therapy to assess changes in blood uremic toxins. Based on this result, future randomized controlled trials will examine the renoprotective effects of sake lees.^125^ These studies would provide novel diets focused on D-AAs for patients with CKD, including DKD. Risperidone inhibits D-AA degradation by regulating DAO activity, which is associated with a decreased risk of kidney function decline in patients with schizophrenia.^58^ Basic and clinical studies are underway to uncover the mechanisms and establish the renoprotective effects of risperidone in patients with and without kidney diseases. An acknowledgment is the current lack of understanding regarding the precise targets and molecular mechanisms of D-AAs and their potential relationship to mitochondrial homeostasis, especially in the context of DKD. Significant efforts are being dedicated to elucidating the role of D-AAs in mitochondrial homeostasis in developing DKD, which may provide novel therapeutic strategies based on this biological axis.

In all, this review provides an overview of mitochondrial disorders, D-AAs, and kidney diseases, with a focus on DKD. This work may introduce innovative approaches and guide future research aimed at effectively controlling and delaying the progression of DKD.

## Disclosure

All the authors declared no competing interests.
